# Self-examination low-cost full-field OCT (SELFF-OCT) for patients with various macular diseases

**DOI:** 10.1007/s00417-020-05035-6

**Published:** 2020-12-21

**Authors:** Claus von der Burchard, Moritz Moltmann, Jan Tode, Christoph Ehlken, Helge Sudkamp, Dirk Theisen-Kunde, Inke König, Gereon Hüttmann, Johann Roider

**Affiliations:** 1 Department of Ophthalmology, University of Kiel, University Medical Center, Arnold-Heller-Strasse 3, 24105 Kiel, Germany; 2 Medical Laser Center Lübeck GmbH, Peter-Monnik-Weg 4, 23562 Lübeck, Germany; 3 University Eye Hospital, Medical School Hannover, Carl-Neuberg-Str. 1, 30625 Hannover, Germany; 4 Institute of Medical Biometry and Statistics, University of Lübeck, Ratzeburger Allee 160, 23562 Lübeck, Germany; 5 Institute of Biomedical Optics, University of Lübeck, Peter-Monnik-Weg 4, 23562 Lübeck, Germany

**Keywords:** Age-related macular degeneration (AMD), Optical coherence tomography (OCT), Biomarker, Home monitoring, Macula, Retina

## Abstract

**Purpose:**

The treatment guidelines for many macular diseases rely on frequent monitoring with optical coherence tomography (OCT). However, the burden of frequent disease control leads to low therapy adherence in real life. OCT home monitoring would address this issue but requires an inexpensive and self-operable device. With self-examination low-cost full-field OCT (SELFF-OCT), our group has introduced a novel technology that may fulfill both requirements. In this pilot study, we report the initial experiences with a clinical prototype.

**Methods:**

Fifty-one patients with different macular diseases were recruited in a cross-sectional study. The most common diseases were age-related macular degeneration (AMD; 39/51), diabetic macular edema (DME; 6/51), and retinal vein occlusion (RVO; 3/51). Patients received a short training in device usage and then performed multiple self-scans with the SELFF-OCT device. For comparison, scans with a standard clinical spectral domain (SD-)OCT were taken.

**Results:**

After a brief training, 77% of the patients were able to successfully acquire images that were clinically gradable. No significant influence on success could be found for age (*p* = 0.08) or BCVA (*p* = 0.97). Relevant disease biomarkers in the most common retinal diseases could be detected.

**Conclusions:**

SELFF-OCT was used successfully for retinal self-examination and in the future could be used for retinal home monitoring. Future improvements in technology are expected to improve success rates and image quality.

**Trial registration:**

The Trial was registered in the German Trial Register under the number DRKS00013755 on 14.03.2018.

**Supplementary Information:**

The online version contains supplementary material available at 10.1007/s00417-020-05035-6.

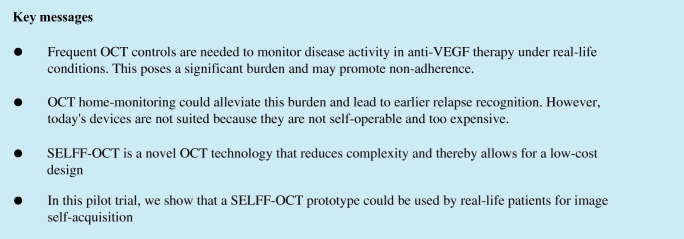



## Introduction

Optical coherence tomography (OCT) is today’s standard retina imaging modality. Because of its non-invasive, fast, and easy application, it can be repeated infinitively and often. Moreover, due to its high resolution and good contrast of retinal layers, it is the most sensitive means of detecting disease activity in many retinal diseases [1] and especially outperforms subjective visual function deterioration [2, 3].

The three most common retinal diseases—age-related macular degeneration (AMD), diabetic macular edema (DME), and retinal vein occlusion (RVO)—can all be effectively treated with intravitreal injection of anti-vascular endothelial growth factor (anti-VEGF) antibodies [4–6]. These treatments usually must be frequently repeated due to disease activity reoccurring [7]. However, it has been shown that the treatment interval can be individualized with the help of frequent OCT controls and still receive comparable outcomes as with a fixed monthly dosing [8]. The introduction of OCT-guided therapy has since become the worldwide standard of care and has saved billions of dollars [9].

Since frequent OCT controls are a key factor to best treatment results [10], frequent office visits are required. By avoiding the necessity of office visits, the development of home-based OCT diagnostics could lower disease burden, improve therapy adherence, and possibly improve the overall treatment outcome. However, current clinical OCT technology is not suited for home monitoring because it is too expensive, too large, and does not allow for patient self-examination. Therefore, we propose a novel compact, extremely low-cost OCT technology: self-examination low-cost full-field OCT (SELFF-OCT). It is based on a special full-field OCT [11] that was designed to cut down device complexity, to reduce component costs and to allow patient self-examination. The device sequentially acquires single-shot en-face images of the retina. In less than a second, a whole volume scan of the central retina can be acquired. By omitting expensive components such as spectrometers, tunable lasers, and scanning systems, this technology has the potential to be built significantly cheaper than current SD- or swept source (SS-)OCT with realistic production costs below US$1000. Moreover, SELFF-OCT can be assembled compactly and robustly. Combined, these advantages make this technology a viable candidate for a home-care scenario. SELFF-OCT has the potential to be manufactured into a hand-held, self-operable device for a few thousand USD retail price.

In this paper, we present the first clinical data of a SELFF-OCT prototype and address the question of both image quality and device self-operability. This first clinical prototype was built as a tabletop device. With this device, patients with various macular diseases performed retinal self-scan without medical personnel assistance.

## Materials and methods

### Technical description of the SELFF-OCT prototype

**Fig. 1:**
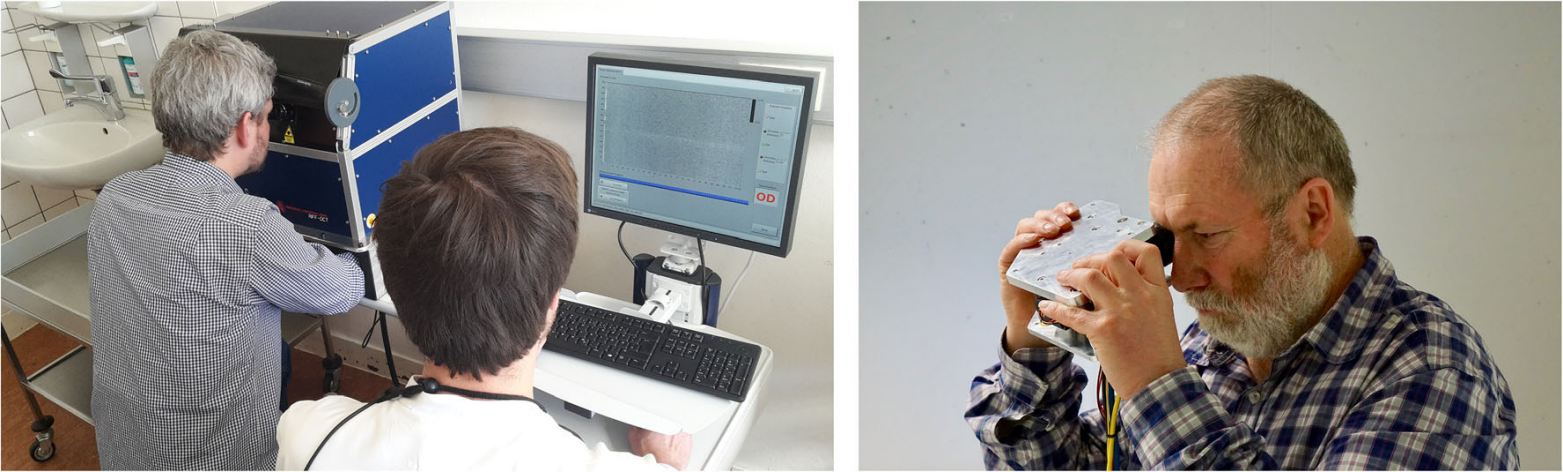
Left: Clinical prototype used for data acquisition within the study. Right: Operational lab prototype with all optical components that shows the potential for device miniaturization

The prototype of our SELFF-OCT uses the principle of off-axis full-field time-domain OCT, which is described in detail by Sudkamp et al. [11]. In short, this design uses an extended illumination of the retina by a 0.9 mW parallel beam from a superluminescent diode (SLD-340-UHP-Toß-PD, Superlum, Cork, Ireland) with a 84-nm wavelength and a 26-nm spectral band width. The retina is imaged onto a CMOS camera, where interference with a slightly tilted reference beam is created. Due to the short coherence length of the light source, only light from a certain depth forms an interference pattern of parallel fringes. This interference pattern is separated from the light scattered in other depths by a Fourier transformation and converted into an en-face image of the retina at that distinctive depth. By rapidly changing the length of the reference arm, the complete thickness of the retina is imaged within less than 1 s. This eliminates the necessity of scanning and other expensive components such as spectrometers or tunable light sources. The system is characterized by its technical simplicity and cost efficiency. Figure 1 (left) shows the clinical prototype used in this study; Fig. 1 (right) shows a working lab prototype that demonstrates the potential to miniaturize the technology.

The investigated SELFF-OCT prototype records a densely sampled volumetric retina scan of a lateral area of 4.5 × 1.4 mm with an axial resolution of 12 μm and a horizontal resolution of about 17 μm. Because the measurement arm can move freely over a distance of 15 mm, the system first performs an overview scan with a lowered axial resolution to locate the RPE and then performs the detailed scans around the RPE with an axial measurement range of 1.4 mm and an axial resolution of 12 μm. Because of this technical feature, the system is able to depict the retina regardless of bulbus length, with possible exceptions in case of extreme ametropia.

### Study protocol

In a prospective clinical study (registered as DRKS00013755 at the German Clinical Trials Register, EUDAMED number CIV-17-12-022384), 51 patients with retinal diseases were recruited to perform a retinal self-scan with the OCT prototype in addition to their routine clinical examination. The study was conducted in accordance with the German Medical Devices Act and the Declaration of Helsinki. Inclusion criteria (above the age of 18 years old and the necessity for macular examination including an OCT scan) were deliberately kept broad in order to depict different diseases in this pilot trial. However, we mainly recruited consecutive AMD patients in order to test the device in the intended target group. One eye was selected as the study eye; only this eye was evaluated in this paper. Main exclusion criteria were significant opacities in the optical media and ametropia greater than ± 3 diopters (range of the diopter adjustment of the prototype). Opacities in the optical media were rated at the physician’s discretion; however, no patient in screening was excluded for this reason, e.g., because of advanced cataract. We further excluded patients with decimal VA under 0.1 in the study eye or obvious difficulty in steady head positioning. No further pre-selection of patients (e.g., fixation testing or geographic atrophy assessment) was undertaken in order to minimize inclusion bias.

After giving informed consent, the patients received a complete assessment of best-corrected visual acuity (BCVA), intraocular pressure (IOP), and a complete examination of the anterior eye segment. Afterward, the patients were introduced to the usage and handling of the SELFF-OCT. This was achieved by an oral introduction from the examiner which lasted only a few minutes. No further training documents or videos were necessary. In short, the patient had to look into the eyepiece mounted on the device. An adjustable headrest could be used for comfortable head positioning during and in-between measurements. Within the eyepiece, a small, green fixation target was presented in order to guide the patient’s eye into correct alignment. Diopter adjustment, if necessary, could be done with a control knob on the eyepiece.

Once the patient was properly adjusted, he started the measurement himself by pushing a hand-held trigger button. During measurement, the patient saw the green fixation target superimposed on the red illumination of the retina by the superluminescent diode. The patient had to keep the fixation target centered on the illumination, which could be done via small head movements.

**Fig. 2:**
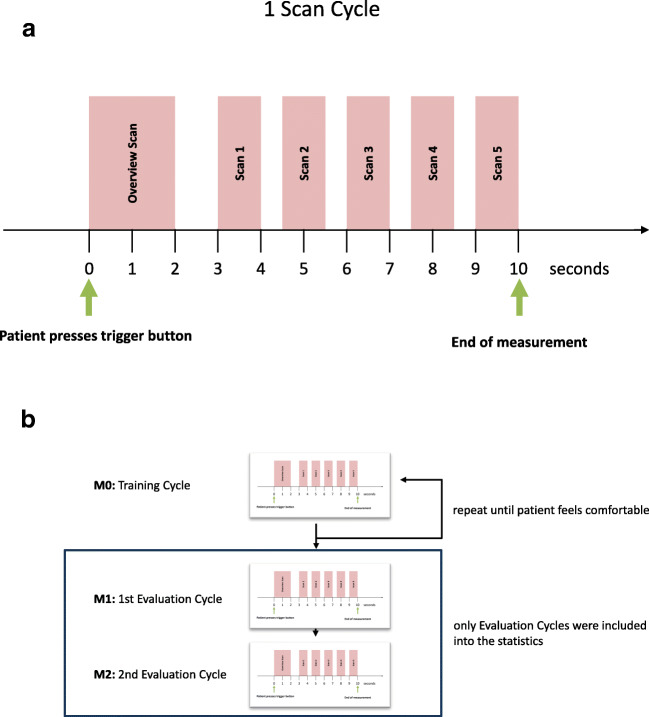
**a** Overview of one scan cycle lasting 10 s. **b** Overview of the measurement protocol

Within one measurement cycle of 10 s, the device first performed one overview scan to locate the exact position of the retinal pigment epithelium (RPE) and then performed five consecutive detailed scans around the RPE that lasted 0.9 s each (Fig. 2a).

Throughout the study protocol, the patient performed several of these measurement cycles. The study protocol included both training cycles and evaluation cycles; only the latter were used for analysis (Fig. 2b).

The first measurement cycle M0 (training cycle) was used as an introduction into the technology and was performed under supervision of the attending physician. During this period, the physician guided the patient into correct head positioning. In contrast to the following measurement cycles, the M0 measurement could be repeated ad libitum, until both the patient and examiner felt certain that the patient was sufficiently experienced with the operation of the device.

Afterward, the patient performed two entire measurement cycles (M1 and M2) without medical assistance. These measurements were included into statistical analysis, regardless of quality or performance. They could not be repeated. Only the completely unassisted measurements were evaluated. All measurements were taken without prior installment of mydriatic eye drops. After finishing the SELFF-OCT measurements, all patients received a detailed scan by a reference OCT (Heidelberg Spectralis HRA+OCT2, 6 × 6 mm volume scan with 49 adjacent B-scans, without enhanced depth imaging (EDI) mode), a color fundus photography (Zeiss FF450plus), and a complete binocular funduscopic examination. Finally, all patients were asked if they were blinded by the measurement or experienced any other adverse events.

### Image rating and overall quality score calculation

The acquired images were processed by the software packages MatLab (Mathworks, Natick, MA, USA) and ImageJ [12]. To increase signal-to-noise ratio (SNR), a floating average filter over 5 adjacent B-scans was applied (Image J Mean 3D filter). Afterward, all images were rated for image quality in a standardized manner. First, one author (M.M.) scored all images (blinded to the reference SD-OCT image) in a dichotomic yes/no grading if the image quality seemed likely to allow for clinical diagnosis. In order to further standardize the grading process and reduce subjectivity, a second scoring system was devised that rated image acquisition artifacts in five defined criteria: motion artifacts, saturation artifacts, vignetting, blurring, and signal strength of the neuroretina. For each criterion, a score between 0 (no artifacts) and 3 (heavy artifacts) was assigned. The ranking was purely based on these artifacts and did not consider the presence of disease-specific biomarkers. The same author once again rated all images for these criteria, blinded to the prior rating. All images where any criterion was marked grade 3 (heavy artifacts) or more than 3 criteria were marked grade 2 (medium artifacts) were rated unsuccessful. Overall, this score compared favorably with the first subjective score and allows for more objective classification of future images. The second scoring system was used to determine scan successfulness in this paper. A detailed explanation of image artifacts and the scoring system can be found in the supplement.

### Data handling and processing

All image data was converted into TIFF files. ImageJ [12] was used for all further image processing. Numerical data was stored in Microsoft Excel. R [13, 14] was used for statistical analysis. For calculations regarding visual acuity such as averaging, VA was converted in logMAR for the calculations and then converted back to decimal for better interpretability. Differences in age and visual acuity between patients who did or did not perform a self-scan were evaluated using Mann-Whitney *U* tests.

## Results

### Study population

In total, 51 study eyes of 51 patients were included into the study. Among these patients, 39 had AMD, 6 DME, 3 branch or central RVO, 1 central serous chorioretinopathy, 1 epiretinal gliosis, and 1 central retinal artery occlusion. Patient age ranged from 45 to 86 years old (mean of 72 years old). BCVA in the study eye was between decimal 0.1 (20/200) and decimal 1.0 (20/20) with a mean of decimal 0.45 (20/45). Twenty-seven patients were pseudophakic, and the remaining ones were phakic. Twenty-three patients were female. Spheric equivalent of the refraction in the study eye ranged from − 3 to + 2.5 with a mean of − 0.23. Age and BCVA broken down according to the diagnosis can be found in Table 1.

**Table 1:** Study demographics for different diseases. Differences in success rate must be interpreted carefully because of the small sample size. Therefore, no statistical testing was performed

Disease	Number	Age			BCVA (decimal)			Success rate
		Min	Max	Mean	Min	Max	Mean	
All	51	45	86	72	0.1	1	0.45	76.5% (39)
AMD	39	48	86	75	0.1	1	0.43	74.4% (29)
DME	6	45	79	64	0.2	0.8	0.38	83.3% (5)
RVO	3	49	84	70	0.6	0.8	0.7	100% (3)
Others	3	47	68	55	0.6	1	0.9	66.7% (2)

No adverse events were recorded during the study.

### Success rate in image self-acquisition

Overall, 48 out of 51 patients (94.1%) were able to acquire a retinal image regardless of image quality. The remaining 3 patients were not able to successfully align their head position, and no retinal structures could be found in their scans.

**Fig. 3:**
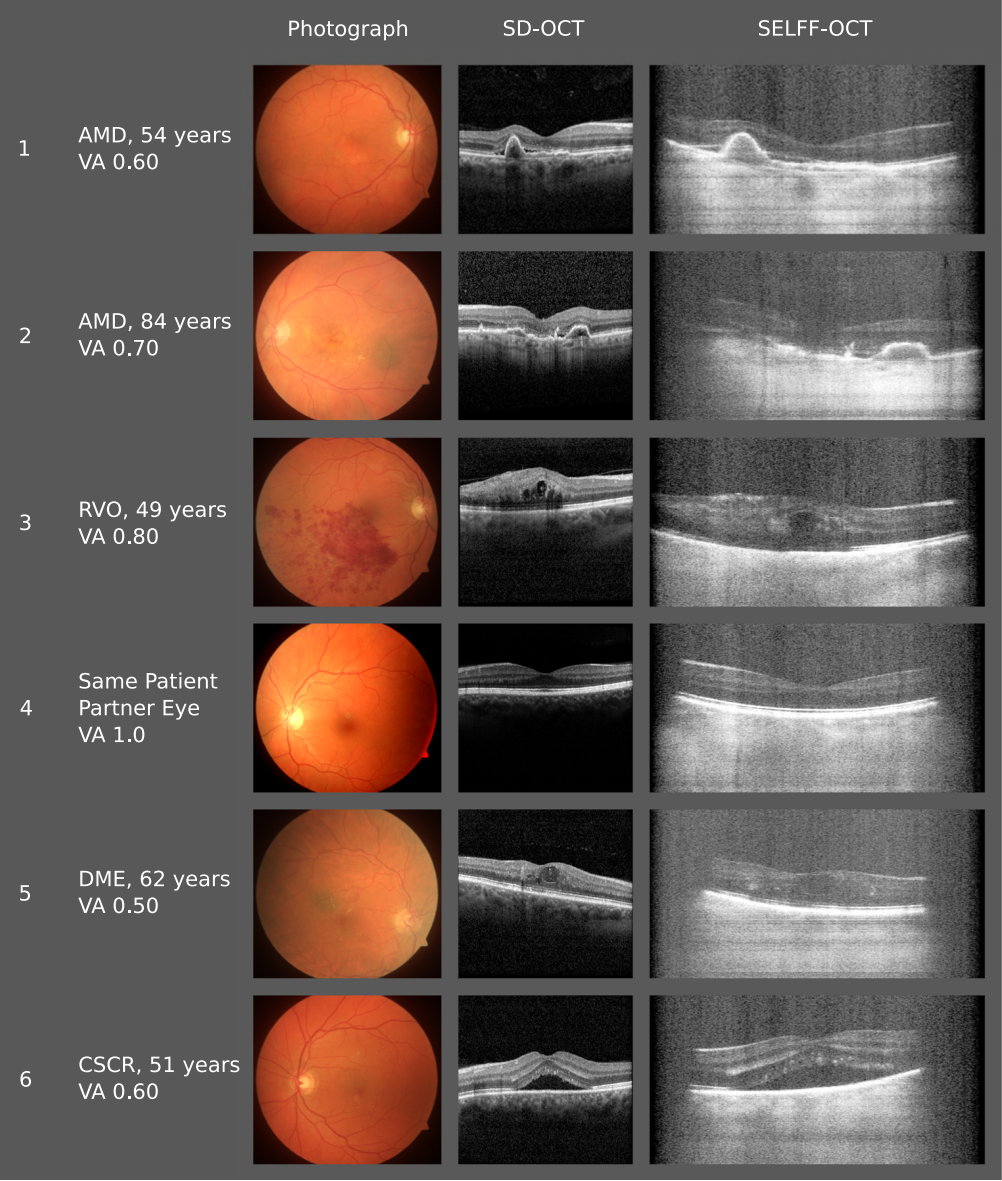
Exemplary SELFF-OCT images from different patients taken with an undilated pupil. Subretinal fluid (SRF) can be found in patients 1, 2, and 6; intraretinal fluid (IRF) in patients 3 and 5; and pigment epithelium detachment (PED) in patients 1 and 2. In patients 5 and 6, vignetting of the outer borders can be noted

Afterward, all images were scored for clinical interpretability as described in the “Materials and methods” section. Thirty-nine out of 51 patients (76%) were able to successfully acquire at least one scan that met the scoring criteria in the study eye. Figure 3 shows representative fundus and SD-OCT and SELFF-OCT images. The main reason for failing scoring criteria was motion artifacts. A detailed discussion of possible artifacts can be found in the Supplement.

### Subanalysis of AMD population

**Fig. 4:**
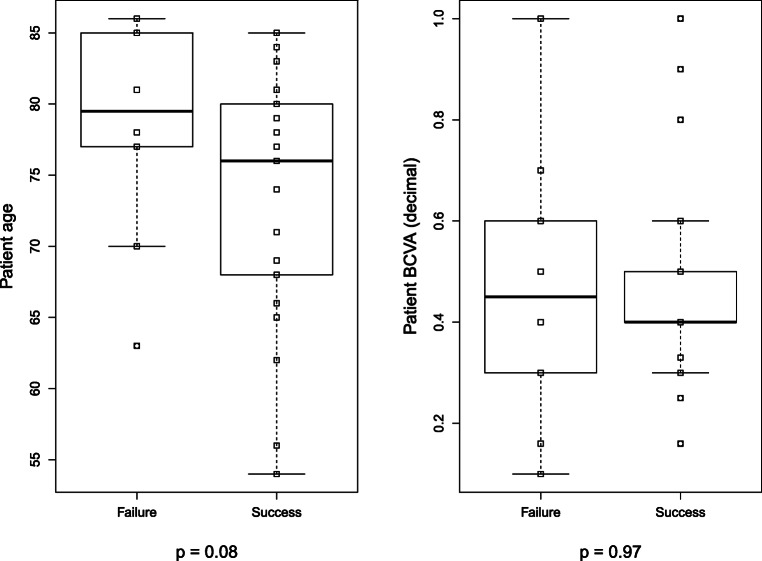
Boxplots comparing success depending on **a** age and **b** BCVA. For both factors, no statistical significance was found

Further subanalysis was performed only for the 39 AMD patients. These were all patients with wet AMD under anti-VEGF therapy or dry AMD patients with suspected wet AMD (i.e., “wet AMD” as referral diagnosis). For other diseases, no sufficient patient numbers were recruited to perform separate subanalysis. It was found in AMD that patients who failed to perform a self-scan tended to be older (Fig. 4a). However, this was not statistically significant (*p* = 0.08; Mann–Whitney–Wilcoxon U-test). Visual acuity did not show any statistically significant difference (Fig. 4b; *p* = 0.974).

### Image quality

Despite higher background noise in SELFF-OCT, the retinal layers were well defined in most cases. Also, the most prominent biomarkers for AMD could be demonstrated: subretinal fluid (SRF; Fig. 3: patients 1, 2, and 6), intraretinal fluid (IRF, Fig. 3: patients 3 and 5), and pigment epithelium detachment (PED; Fig. 3: patients 1 and 2). Due to vast light scattering of the RPE, visibility below the RPE into the choroid is heavily limited compared with SD-OCT, so that apart from the Bruch’s membrane, no further details were distinguishable there. In the patient collective included in this study, no significant image quality deterioration was seen in cataract patients.

## Discussion

We conducted this study to assess both the success rate of a self-operable OCT device in the target population and to investigate the image quality of this new technology in a clinical setting. The image resolution was close to the reference OCT but had a poorer signal-to-noise ratio and additional artifacts. The reduced image quality is a consequence of the low-cost concept. The main disadvantage of the design is the influence of reflected and numerously scattered light. This is due to abandoning the scanning of the retina, as it is also observed with fundus cameras compared with laser scanning ophthalmoscopy (SLO). In contrast to SD-OCT, only light from one depth is measured in one exposure, which reduces the sensitivity. Current technical limitations are the limited output power of the light source and the readout frequency of the camera [11], which lead to a low signal-to-noise ratio and motion artifacts, respectively. In addition, the device used here suffered from spurious reflections caused by the numerous surfaces of the optical components, which cause visible background noise in the images and saturation artifacts. These reflections will be reduced in future devices through better anti-reflection coating and an optimized optical layout. We recently showed that ametropia correction can be accomplished numerically [15]. This further simplifies the design, cuts down costs, and reduces artifacts.

Since the device is not intended for regular ophthalmic diagnosis, but only for monitoring disease activity, reduced image quality is acceptable as long as morphological changes can be detected with sufficient sensitivity and specificity. Furthermore, mere monitoring of central retinal volume has been shown to be sensitive in AMD monitoring, which could be used as a further monitoring criterion [16]. In case of disease activity, the device would refer the patient to his doctor for a confirmatory OCT scan and possibly an immediate intravitreal injection in case of confirmed activity.

In our study, we were able to detect all common biomarkers of disease activity in AMD, DME, and RVO, including PED, SRF, IRF, intraretinal hyperreflective foci, and intraretinal hemorrhage (see Fig. 3). SRF is easily detectable since both the adjacent RPE and ellipsoid zone are hyperreflective. In contrast, IRF does not have hyperreflective borders and is therefore more challenging to detect. However, it could still be visualized with SELFF-OCT.

The technical term full field, established to describe the simultaneous illumination of the scan area as opposed to scanning OCT systems, should not be confused with the size of the scan area. Quite the contrary, our scan size (4.5 × 1.4 mm) is limited by the specifications of low-cost CMOS cameras and smaller compared with most SD-OCT scanning protocols. However, a theoretical study showed that already small central OCT scans of 2 mm have high sensitivity in detecting disease activity [17]. However, this will need confirmation in future clinical studies.

### Device ergonomics

In contrast to current OCT devices, the investigated SELFF-OCT allows unassisted OCT self-examination. To achieve correct alignment, the patient has to center the fixation target into the center of the illuminated part of the retina, which the patient sees as a red circular area. This creates a “keyhole” effect and automatically guides the patient into the correct position. Scanning OCT systems would require additional optics to achieve a similar effect for a self-alignment, which would further increase overall cost, device size, and complexity.

The keyhole alignment method proved to be very reliable even in patients with low BCVA. These patients, in general, tended to need more tries in the training and longer time to locate the fixation target, but overall success rates did not differ. However, it limits device usage to patients with some residual visual function. Especially in geographic atrophy, usability may be limited. For other patients, variations in the fixation target, e.g., a circle or star, could possibly further reduce the problem. Also, we found that the gel cushion headrest we used in our study offered too little head stability. In the future, more rigid headrest designs are expected to significantly lower motion artifacts. In-lab testing with a handheld device (Fig. 1b) showed that because of improved patient interface, less motion artifacts occurred than with the tabletop device, despite it being handheld.

Nevertheless, because of the requirement for self-alignment and the necessity of keeping still during measurements, SELFF-OCT has minimum requirements concerning cognitive function, visual function, and musculoskeletal function that are less relevant in clinical OCT systems. Therefore, we excluded patients who would definitely not meet these requirements, since they would also not be eligible patients for home monitoring. However, we did not perform further extensive pre-selection of the patients such as fixation testing, but rather included a broad real-life cross-section of AMD patients. With these givens, more than three quarters of the patients (77%) were able to acquire images that were clinically gradable. While this leaves room for improvement, we consider this success rate encouraging for a pivotal trial. In our belief, if a similar success rate as the 77% in this study could be found in a later real-world home-care scenario and 3 out of 4 presumable candidates could be monitored with home-care OCT, this success rate would indeed be very satisfactory. On top of that, we are confident that the success rate can be improved in future devices.

### Motivation for home-care devices

In all diseases where regular OCT monitoring is necessary, this comes with high disease burden for the patients. For AMD, most guidelines propose monthly monitoring even when no disease activity has been present for longer periods of time [4, 18]. These frequent monitoring visits significantly stress out patients and lead to low therapy adherence [10, 19]. Not only are frequent follow-up visits time-consuming, but because the patients are mostly elderly and often physically impaired, they often require assistance from relatives or other people. A long distance between the patient home and the ophthalmologist was found to be the leading cause of therapy non-adherence [19]. Moreover, frequent visits bind the capacities of healthcare professionals and create considerable healthcare costs.

Low patient adherence leads to significantly reduced treatment outcome [10]. Efforts to alleviate some of the treatment burden were undertaken, e.g., with the introduction of the treat-and-extend (TAE) dosing regimen [20]. TAE tries to predict the relapse interval and adapts treatment schedules accordingly. Additionally, it combines monitoring and treatment visits. While this can reduce the amount of overall patient visits, it can also create both disease overtreatment [21] and undertreatment in case of an early disease relapse.

The introduction of a sensitive home monitoring system for retinal diseases would address all these issues by allowing daily disease monitoring at a minimal additional burden to the patient. Moreover, it would allow for tailor-made individualized therapy, eliminate overtreatment or undertreatment, and guarantee the best possible treatment outcomes. Therefore, an OCT-based home monitoring system could lead to a completely new monitoring and treatment scheme for these patients.

### Comparison with similar approaches

Other research groups have also addressed the question to make OCT home-care compatible [22–24]. Recently, a design for a low-cost spectral domain OCT (SD-OCT) with component costs of below US$6000 was published [22]. However, self-examination was not tested, and OCT scans were acquired with a dilated pupil. Therefore, it is unknown how suitable the device would be for self-examination. Currently, a home-care OCT solution is under testing by the company Notal Vision [24]. Their device uses conventional SD-OCT technology and is also not expected to be cheaper than the low-cost OCT published [22]. Typically, medical devices are sold with more than twice to three times the component costs. Without technological breakthroughs, like fully integrated OCT on one optical chip [25], we see no further price reduction potential for SD- or SS)-OCT. However, device cost is crucial for the concept of home monitoring because in contrast to an OCT device in a clinical environment, the home device will only be used a few minutes per day.

### Outlook

Regardless of underlying technology, home monitoring of AMD progression by OCT requires an efficient and cost-effective image interpretation. Analyzing the recorded volumes by the care-taking ophthalmologist or some other professional seems impracticable because of the excessive number of images which are generated by daily self-examination. With the recent advancements in computer science, artificial intelligence (AI) seems to be the most promising option to solve this problem [26]. Especially with the limited quality of home-care OCT systems, it is conceivable that AI trained on both SD- and SELFF-OCT might even outperform human readers. Consequently, we are currently developing AI algorithms for interpretation of SELFF-OCT images [27, 28].

Home monitoring OCT has huge potential to create a paradigm shift in ophthalmic healthcare with benefits for the patients, doctors, and public health. Currently, we are working on a portable low-cost version of the SELFF-OCT technology, which patients will use at home for conducting a longitudinal study on the value of home monitoring by OCT.

## Supplementary information

ESM 1 (DOCX 2399 kb)

## Data Availability

Not applicable.
