# Supplementary material for: Self-examination low-cost full-field OCT (SELFF-OCT) for patients with various macular diseases
Source: Graefes Arch Clin Exp Ophthalmol. 2020 Dec 21;259(6):1503–11. doi: 10.1007/s00417-020-05035-6 (PMC8166739; doi:10.1007/s00417-020-05035-6)
Supplement: Supplementary file 1 — (DOCX 2399 kb) [file 417_2020_5035_MOESM1_ESM.docx]

Self-Examination Low-Cost Full-Field OCT (SELFF-OCT) for Patients with Various Macular Diseases

# Supplement: Explanation of the scoring system

Each image obtained by the SELFF-OCT was rated for five criteria, which were each assigned a score between 0 and 3. Examples can be found in Figure 1.

- Motion Artifacts (horizontal black bars within the picture):
  - 0: no motion artifacts
  - 1: motion artifacts outside of the neurosensory retina or minimal artifacts within
  - 2: small motion artifacts inside the neurosensory retina that are too small to cover any significant structures
  - 3: large artifacts that destroy the overall usability of the picture
- Saturation Artifacts:
  - 0: no saturation artifacts
  - 1: small saturation artifacts at the peripheral image area that maximally cover a tiny fraction of the image
  - 2: medium saturation artifacts that are too small to cover significant retinal structures
  - 3: heavy saturation artifacts that destroy the overall usability of the picture
- Vignetting:
  - 0: no vignetting
  - 1: small vignetting of the peripheral field of view. Fovea is centered and well detectable
  - 2: medium vignetting artifacts. Greater peripheral vignetting. The fovea is possibly excentric, but still completely within the picture
  - 3: Large vignetting that includes the fovea.
- Blurring:
  - 0: Interal limiting membrane (ILM) is sharp. In the outer layers, the ellipsoid zone, the interdigitation zone and the retinal pigment epithelium are three discriminable hyperreflective layers
  - 1: The interdigitation zone and retinal pigment epithelium are blurred and possibly merged. The ellipsoid zone is well demarkable
  - 2: Ellipsoid zone is blurred and not well demarkable
  - 3: Ellipsoid zone is not detectable
- Signal Strength of Neuroretina:
  - 0: The main retinal layers are well-detectable and have a high contrast
  - 1: The main retinal layers are detectable, but with lowered contrast
  - 2: The main retinal layers are hardly detectable and discriminable
  - 3: The main retinal layers are not discriminable

Afterwards, an overall score was combined out of the single criteria. All cases where one ore more criteria was marked as heavy (criterion score 3), the overall score was set too 100. Otherwise, non-existing artifacts (criterion score 0) were assigned 0 points, small artifacts (criterion score 1) 2 points and medium artifacts (criterion score 2) 20, and the overall score was a summation of the five assigned points for the scoring criteria.


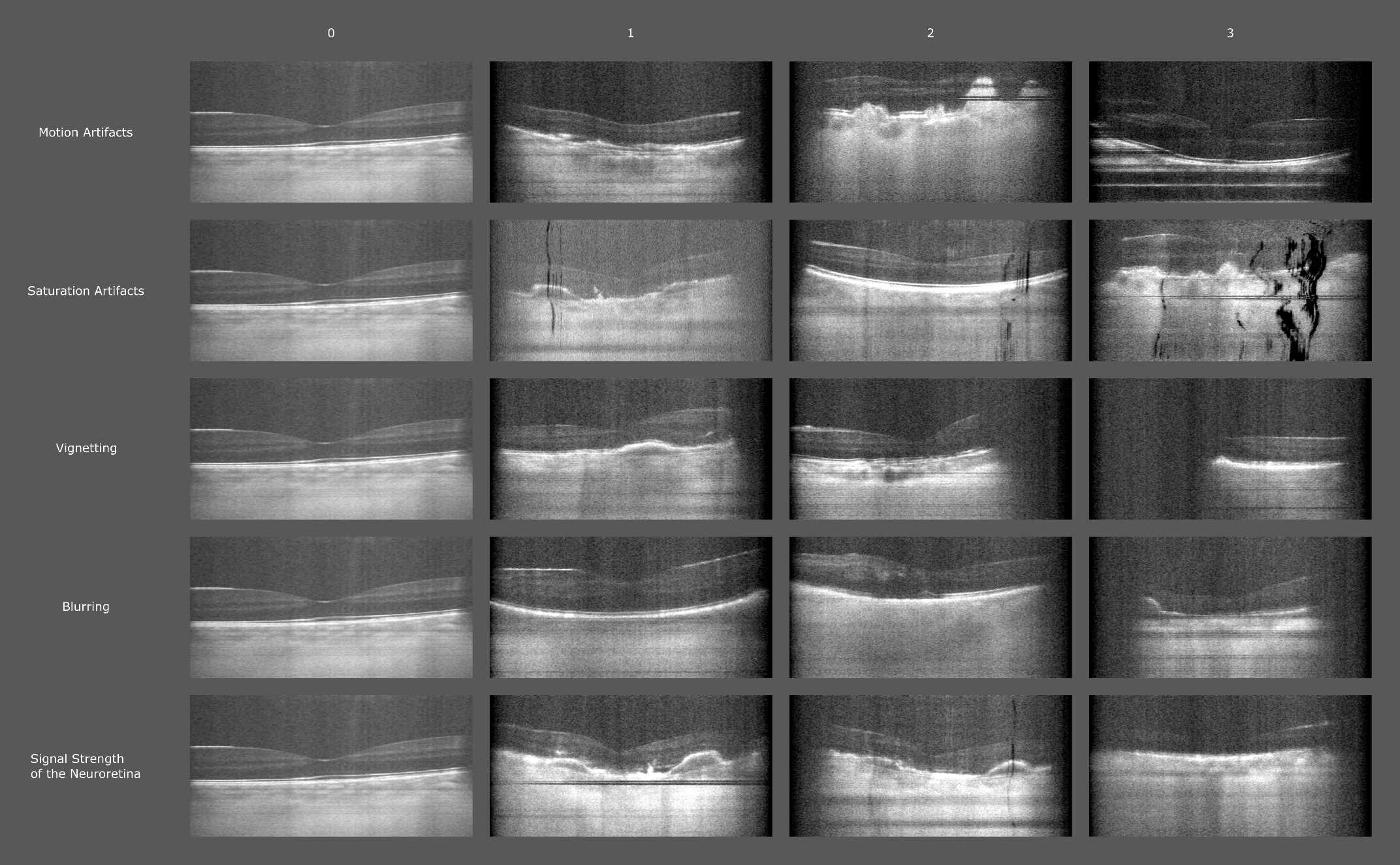


Figure 1: Scoring matrix with examplery images for each score for each criterion.
